# Continued concomitant antibiotic or immunosuppression therapies reduce the risk of pouchitis among patients with ileal pouch-anal anastomosis and primary sclerosing cholangitis

**DOI:** 10.1093/crocol/otag091

**Published:** 2026-08-04

**Authors:** John P Haydek, Priscila Santiago, Joseph Carter Powers, Latchmin Ruplall, Hoang Anh Richardson, Erin Causey, Mohamed Elhawary, Taylor Karl, Rami Tfayli, Jana G Hashash, Taha Qazi, Shannon Chang, Sara Horst, Frank I Scott, Millie D Long, Laura Raffals, Edward L Barnes

**Affiliations:** Division of Gastroenterology and Hepatology, University of North Carolina, Chapel Hill, NC, United States; Division of Gastroenterology and Hepatology, Mayo Clinic, Rochester, MN, United States; Division of Gastroenterology, Cleveland Clinic, Cleveland, OH, United States; Division of Gastroenterology and Hepatology, NYU Langone Health, New York, NY, United States; Division of Gastroenterology, Hepatology, and Nutrition, Vanderbilt University, Nashville, TN, United States; Division of Gastroenterology, Hepatology, and Nutrition, Vanderbilt University, Nashville, TN, United States; Division of Gastroenterology and Hepatology, NYU Langone Health, New York, NY, United States; Division of Gastroenterology and Hepatology, University of Colorado School of Medicine, Aurora, CO, United States; Division of Gastroenterology and Hepatology, Mayo Clinic, Jacksonville, FL, United States; Division of Gastroenterology and Hepatology, Mayo Clinic, Jacksonville, FL, United States; Division of Gastroenterology, Cleveland Clinic, Cleveland, OH, United States; Division of Gastroenterology and Hepatology, NYU Langone Health, New York, NY, United States; Division of Gastroenterology, Hepatology, and Nutrition, Vanderbilt University, Nashville, TN, United States; Division of Gastroenterology and Hepatology, University of Colorado School of Medicine, Aurora, CO, United States; Division of Gastroenterology and Hepatology, University of North Carolina, Chapel Hill, NC, United States; Center for Gastrointestinal Biology and Disease, University of North Carolina, Chapel Hill, NC, United States; Division of Gastroenterology and Hepatology, Mayo Clinic, Rochester, MN, United States; Division of Gastroenterology and Hepatology, University of North Carolina, Chapel Hill, NC, United States; Center for Gastrointestinal Biology and Disease, University of North Carolina, Chapel Hill, NC, United States

**Keywords:** pouchitis, primary sclerosing cholangitis, chronic antibiotic refractory pouchitis, chronic antibiotic–dependent pouchitis, Crohn’s-like disease of the pouch

## Abstract

**Background:**

Complications such as intermittent pouchitis (IP) and chronic inflammatory disorders of the pouch (CP) are common after ileal pouch-anal anastomosis (IPAA), particularly among patients with both ulcerative colitis and primary sclerosing cholangitis (PSC). There are no standardized approaches for prophylaxis against these complications for patients with PSC undergoing IPAA. We compared outcomes among patients with PSC and IPAA who received antibiotic or immunosuppressive therapies to those who did not.

**Methods:**

We designed a retrospective cohort study that included patients with ulcerative colitis and PSC from 7 tertiary care centers that underwent IPAA between 1994 and 2023. Patients were categorized on whether they received medications following IPAA that may provide primary prophylactic benefit, such as antibiotics, probiotics, immunomodulators, or biologic medications; and the proportion of patients developing IP and CP was evaluated.

**Results:**

A total of 172 patients were included in the cohort, and 13 (7.6%) patients received antibiotics or immunosuppressant medications. Antibiotics and probiotics were the most common therapy (5 patients, 2.9%), followed by biologics, small-molecule inhibitors, immunomodulators, or mesalamine (all 1 patient, 0.6%). Compared to patients not receiving antibiotics or immunosuppression, patients receiving antibiotics or immunosuppression were significantly less likely to develop IP (69.2% vs 91.2%; *P* = .03; odds ratio 0.22; 95% CI, 0.06-0.80) and CP (23.1% vs 56.0%, *P* = 0.04; OR 0.24, 95% CI, 0.06-0.89).

**Conclusions:**

In this retrospective cohort of patients with ulcerative colitis and PSC undergoing IPAA, usage of concomitant antibiotics or immunosuppression was low. However, use of concomitant antibiotics or immunosuppression may decrease the burden of inflammatory conditions of the pouch in these high-risk patients.

## Introduction

Despite advances in the medical treatment of ulcerative colitis (UC), approximately 13% of patients will ultimately require a colectomy due to refractory disease or dysplasia.^1^ Although restorative proctocolectomy with ileal pouch-anal anastomosis (IPAA) removes the colon thereby removing the driver of UC, serious complications can occur including intermittent pouchitis (IP) and chronic inflammatory conditions of the pouch (CP), composed of chronic antibiotic dependent pouchitis (CADP) and chronic antibiotic refractory pouchitis (CARP), and Crohn’s-like disease of the pouch (CLDP).^2^ Patients with a preoperative diagnosis of primary sclerosing cholangitis (PSC) are at a particularly high risk for developing IP and CP when compared to patients with UC without PSC undergoing IPAA.^3^

Despite their unique risk of IP and CP, there are no standardized approaches for the treatment of patients with PSC undergoing IPAA. Among all patients undergoing IPAA, the American Gastroenterological Association recently published clinical practice guidelines on the management of pouchitis and inflammatory pouch conditions but recommended against antibiotics as a method of primary prophylaxis and made no recommendation for or against usage of probiotics for primary prevention, primarily on the basis of a lack of data from high quality clinical trials.^4^ Given these gaps, we performed a retrospective study evaluating the impact of concomitant therapies continued from the time of the final stage of IPAA surgery as an indicator of the potential role of primary prophylaxis against pouchitis among patients with PSC who undergo IPAA.

## Methods

We performed a retrospective cohort study of adults (≥18 years old) with UC and PSC who underwent IPAA creation and received care at 1 of 7 tertiary healthcare systems between January 1994 and May 2023. For inclusion, patients were required to have a diagnosis of UC and PSC based on International Classification of Diseases, Ninth Revision (ICD-9) or International Classification of Diseases, Tenth Revision (ICD-10) codes, a previously validated method.^5^ Alternatively, patients could have a clinical diagnosis of PSC indicated by their treating physician, which was identified on review of the medical record.

### Primary exposure

Any continued prescription of the following medications from the time of the final stage of IPAA surgery was analyzed as a concomitant antibiotic or immunosuppression: antibiotics, probiotics, 5-aminosalicylates, biologics, immunomodulators, or small molecules. All medications were analyzed for continued use from time of ileostomy takedown or the final stage of IPAA surgery. A medication was considered a continued prescription if it was documented as ongoing at the time of the final stage of IPAA surgery with no minimum duration threshold defined. The indication for each medication was not systematically captured due to the retrospective nature of the study.

### Primary outcome

The diagnosis of IP, CP, and CLDP were based on the discretion of the treating physician. The definitions of IP, CP, and CLDP were based on the definitions used in recent national guidelines on pouchitis and inflammatory pouch disorders.^4^ While endoscopic findings can support an underlying diagnosis, endoscopy was not required for a diagnosis of IP, CP or CLDP.

Our primary outcome was the proportion of patients that developed IP. Our secondary outcome was the proportion of patients that developed CP, which were considered present if a patient was clinically diagnosed with CARP, CADP, or CLDP. Records were collected following the final stage of IPAA surgery until the diagnosis of CP. If CP did not occur, the last follow-up date was recorded. Covariables of interest were manually extracted from the electronic medical record.

Demographic information, clinical outcomes, and exploratory analyses were summarized using descriptive statistics. Fisher’s exact test was used to compare outcomes between groups. Univariable logistic regression was used to calculate odds ratios. Time-to-event analysis was performed using Kaplan-Meier curves to assess comparative time-to-diagnosis and univariable Cox proportional hazard models for associations between prophylaxis use and time-to-outcomes. Censoring was employed at time of CP diagnosis or last date of follow-up, whichever happened first. All statistical analysis was performed using Stata BE 18.5 (College Station, Texas). This research was approved by the University of North Carolina’s Office of Human Research Ethics and the corresponding Institutional Review Board at each participating center.

## Results

We identified 172 patients with UC and PSC who underwent IPAA within the study period (Table 1), of whom 13 (7.6%) received concomitant antibiotics or immunosuppression. Among all advanced therapies used prior to IPAA creation, infliximab was the most frequently used (50.6% of patients exposed, Table 2). Of the available data, 12.1% of patients were exposed to ursodiol within 3 months of colectomy; 12.8% exposed within 3 months of IPAA creation; and 12.2% exposed at time of final IPAA surgical stage.

**Table 1:** Demographic and clinical characteristics among patients with primary sclerosing cholangitis and ulcerative colitis undergoing restorative proctocolectomy with ileal pouch-anal anastomosis.

Variable		Patients undergoing IPAA *N* = 172 (%)
**Age, median (SD)**		40.5 (13.8)
**Sex**	Male	113 (65.7)
	Female	59 (34.3)
**Race**	White	148 (94.3)
	Black	7 (4.5)
	Asian	2 (1.3)
**Hispanic**	Yes	10 (6.3)
	No	149 (93.7)
**Disease extent of ulcerative colitis prior to IPAA**	Proctitis	1 (0.7)
	Left-sided	5 (3.6)
	Extensive	133 (95.7)
**Stages of IPAA**	I	2 (1.3)
	II	54 (34.2)
	Modified II	3 (1.9)
	III	99 (62.7)
**Smoking status**	Current	4 (2.4)
	Former	22 (12.9)
	Never	144 (84.7)
**IPAA indication**	Medically refractory disease	98 (57.0)
	Dysplasia/cancer	56 (32.6)
	Other	5 (2.9)
	Both dysplasia/cancer and medically refractory disease	13 (7.6)
**Any non-PSC extraintestinal manifestations**	Yes	32 (18.6)
	No	140 (81.4)
	Oral steroids	133 (88.1)
**Concomitant antibiotic or immunosuppression**	Yes	13 (7.6)
	No	159 (92.4)
**Type of concomitant therapy**	Antibiotics	5 (2.9)
	Probiotics	5 (2.9)
	Biologics	1 (0.6)
	Small molecule	1 (0.6)
	Immunomodulator	1 (0.6)
	5-aminosalicylates	1 (0.6)

Percentages may not add up to 100% due to unknown exposure history for some patients.

**Table 2:** Medication exposure prior to colectomy and ileal pouch-anal anastomosis for ulcerative colitis.

Medication		*N* (%)
**Topical aminosalicylates**		26 (18.2)
**Oral aminosalicylates**		138 (88.5)
**Thiopurines**		84 (54.6)
**Methotrexate**		9 (6.0)
**Anti-tumor necrosis factor**	Any	100 (62.9)
	Adalimumab	37 (21.5)
	Certolizumab pegol	5 (2.9)
	Golimumab	4 (2.3)
	Infliximab	87 (50.6)
**Vedolizumab**		41 (24.7)
**Ustekinumab**		8 (4.9)
**Tofacitinib**		9 (5.5)
**Topical steroids**		16 (11.0)
**Oral steroids**		133 (88.1)

Percentages may not add up to 100% due to unknown exposure history for some patients.

Among 173 included patients, IP occurred in 154 (89.5%). CP occurred in 92 patients (53.5%), with 52 patients diagnosed with CADP (30.2% of all patients), 10 patients diagnosed with CARP (5.8% of all patients), and 30 patients diagnosed with CLDP (17.4% of all patients).

IP was significantly less likely to occur among patients utilizing concomitant antibiotics or immunosuppression when compared to patients who did not utilize concomitant therapies (69.2% vs 91.2%; *P* = .03; odds ratio [OR] 0.22; 95% CI, 0.06-0.80). Due to small sample sizes in each treatment category, individual statistical evaluations were not possible for the majority of therapy types, although use of antibiotics was associated with decreased odds of IP (OR 0.14, 95% CI, 0.02-0.94). CP was also significantly less likely to occur among patients utilizing concomitant antibiotics or immunosuppression (23.1% vs 56.0%, *P* = .04; OR 0.24, 95% CI, 0.06-0.89). When CARP, CLDP, and CLDP were analyzed individually, there were no statistical associations.

The median time to development of IP was 376 days (interquartile range [IQR], 927 days). There were no differences in time-to-IP (313 days vs 376 days, *P* = .40) nor time-to-CP (480 days vs 1105, *P* = .20), when comparing those who received concomitant antibiotics or immunosuppression and those who did not. There was no significant difference when comparing the median time to either a diagnosis of CP or last follow-up date among those patients receiving concomitant antibiotics or immunosuppression (1852 days, IQR 2365) to those with no concomitant therapy (median 1357 days, IQR 2955; *P* = .85).

Given the relative difference in follow-up time between the 2 groups, a time-to-event analysis was performed using a 5-year window from time of IPAA creation. A univariable Cox proportional hazards model was generated for IP and CP evaluating whether the use of concomitant therapy affected the time-to-diagnosis. These models were not significant for IP (HR 0.76, 95% CI, 0.38-1.50) or CP (HR 0.48, 95% CI, 0.18-1.80) in patients receiving concomitant antibiotics or immunosuppression. Given the small sample size, multivariable regression was not performed.

## Discussion

In this observational cohort of patients with UC and PSC undergoing colectomy with IPAA, concomitant therapy with antibiotics or immunosuppression was associated with a significant reduction in the overall proportion of patients developing IP and CP. While comprehensive subgroup analysis was limited due to small sample sizes, use of antibiotics was associated with decreased proportion of IP, a finding previously demonstrated in prospective studies.^6^

We demonstrated a potential benefit of utilizing antibiotics and/or immunosuppression as a method of primary prevention among patients with PSC undergoing IPAA. When considering both primary and secondary prevention strategies, focusing on a high-risk population such as PSC^3^ may offer the highest yield.^7^ In addition to a potentially high-risk clinical phenotype, patients with PSC undergoing IPAA have demonstrated underlying physiologic changes^8^ that may inform both risk prediction and future selection of preventive agents.

In our cohort, the indication for antibiotics or immunosuppression was not analyzed. We recognize the potential for confounding by indication in this retrospective analysis. However, the potential benefits from these medications would extend to IP and CP regardless of indication, especially given the recognition that some patients may have been continued on therapies given a suspected increased risk of pouchitis, thus biasing toward the null. The association between primary prophylaxis use and a reduction in IP and CP was demonstrated in logistic regression but not in Cox regression. There is the potential that longer follow up and the smaller sample size ultimately leads to an underpowered analysis. Epidemiologic data suggests that the risk of developing pouchitis in an individual patient is almost a certainty if that individual is followed long enough.^9^ These increased risks for both IP and CP over time would indicate that patients followed for longer total durations in our cohort of patients with PSC are at a substantially higher risk for developing IP and CP compared to patients with shorter follow-up times. In our analyses, where only a few patients received prophylaxis, this may have limited the ability to demonstrate an effect of prophylaxis with long follow-up times, and this remains an objective for future, larger, prospective studies.

This study has several important limitations. First, medication indications were not systematically captured; prescriptions classified as concomitant therapy may have been initiated for reasons unrelated to pouchitis prophylaxis, such as treatment of other non-pouch-related conditions. This represents a potential for confounding by indication that cannot be fully resolved from retrospective chart review alone. Second, given the retrospective nature of this research, we are unable to confirm medication adherence. Third, the small sample size of patients receiving concomitant therapy precluded subgroup analyses by individual medication class. Probiotics and antibiotics were analyzed together with immunosuppressive agents as composite exposure, which may be mechanistically inappropriate given their distinct modes of action; future studies should evaluate these classes separately. Last, although the study period extends to 2023, enrollment began in 1994, and the therapeutic landscape for IBD has changed substantially during this interval, with many currently available advanced therapies not yet in routine use during earlier enrollment years.

In summary, in a high-risk cohort of patients undergoing IPAA with PSC, we demonstrated the potential impact of concomitant antibiotics and immunosuppression on the development of IP and CP. These retrospective data should prompt future prospective studies evaluating the role of primary and secondary prevention in one of the highest-risk populations undergoing IPAA for UC.

## Data access

Data are not publicly accessible, but available upon reasonable request to corresponding author.

## References

[1] Tsai L , Ma C, Dulai PS, et al Contemporary risk of surgery in patients with ulcerative colitis and Crohn’s disease: a meta-analysis of population-based cohorts. Clin Gastroenterol Hepatol. 2021;19:2031-2045. 10.1016/j.cgh.2020.10.039 33127595 PMC8934200

[2] Barnes EL , Herfarth HH, Sandler RS, et al Pouch-related symptoms and quality of life in patients with ileal pouch-anal anastomosis. Inflamm Bowel Dis. 2017;23:1218-1224. 10.1097/mib.0000000000001119 28426474 PMC5512700

[3] Barnes EL , Holubar SD, Herfarth HH. Systematic review and meta-analysis of outcomes after ileal pouch-anal anastomosis in primary sclerosing cholangitis and ulcerative colitis. J Crohns Colitis. 2021;15:1272-1278. 10.1093/ecco-jcc/jjab025 33544128

[4] Barnes EL , Agrawal M, Syal G, et al; AGA Clinical Guidelines Committee. AGA clinical practice guideline on the management of pouchitis and inflammatory pouch disorders. Gastroenterology. 2024;166:59-85. 10.1053/j.gastro.2023.10.015 38128971 PMC11163976

[5] Hou JK , Tan M, Stidham RW, et al Accuracy of diagnostic codes for identifying patients with ulcerative colitis and Crohn’s disease in the veterans affairs health care system. Dig Dis Sci. 2014;59:2406-2410. 10.1007/s10620-014-3174-7 24817338 PMC6907154

[6] Ha CY , Bauer JJ, Lazarev M, et al Early institution of tinidazole may prevent pouchitis following ileal-pouch anal anastomosis (IPAA) surgery in ulcerative colitis (UC) patients. Gastroenterology. 2010;138:S-69.

[7] Barnes EL , Herfarth H. Primary prevention of pouchitis. Clin Gastroenterol Hepatol. 2025;23:1492-1496. 10.1016/j.cgh.2025.03.008 40451251 PMC13135415

[8] Santiago P , Quinn KP, Chen J, et al Altered bile acid and pouch microbiota composition in patients with chronic pouchitis. Inflamm Bowel Dis. 2024;30:1062-1070. 10.1093/ibd/izad288 38037191 PMC11219471

[9] Barnes EL , Allin KH, Iversen AT, Herfarth HH, Jess T. Increasing incidence of pouchitis between 1996 and 2018: a population-based Danish cohort study. Clin Gastroenterol Hepatol. 2023;21:192-199. 10.1016/j.cgh.2022.04.015 35525393 PMC9636065

